# Brucellosis in Saudi Children: Presentation, Complications, and Treatment Outcome

**DOI:** 10.7759/cureus.11289

**Published:** 2020-11-01

**Authors:** Salman S Qasim, Khalid Alshuwaier, Mohammed Q Alosaimi, Mohammad A Alghafees, Abdullah Alrasheed, Laila Layqah, Salim Baharoon

**Affiliations:** 1 Medicine, King Saud Bin Abdulaziz University for Health Sciences, Riyadh, SAU; 2 Pharmacy, King Abdullah International Medical Research Center, Riyadh, SAU; 3 Internal Medicine, King Abdulaziz Medical City Riyadh, Riyadh, SAU

**Keywords:** brucellosis, brucella melitensis, children, brucella abortus

## Abstract

Background

Brucellosis, an endemic disease in Saudi Arabia, has an infection rate of 70 per 100,000 people, with a varying morbidity rate in different parts of the country. The aim of this study was to assess the epidemiological and clinical features, laboratory findings, treatment modalities, complications, and outcomes in children with brucellosis.

Materials and methods

The medical records of 153 patients attending King Abdullah Specialist Children’s Hospital in Riyadh, Saudi Arabia, from January 2015 to January 2019 were reviewed retrospectively. Demographic data, laboratory test results, serum agglutination test titer, and the results of the blood cultures were obtained. The diagnosis of brucellosis was based on compatible signs and symptoms with a positive serology titer of ≥1:160 or a blood culture positive for *Brucella* species.

Results

The majority of the sample (69.6%, n=107) were males, with a mean age of 7.75 ± 3.28 years. Ingestion of unpasteurized camel dairy products was the most frequent transmission risk factor. The most prevalent presenting symptoms were constitutional and musculoskeletal symptoms. Six patients (3.9%) had complicated brucellosis, with neurobrucellosis diagnosed in three cases. Hospitalization for brucellosis was required in 15% of the patients. The majority (99.35%, n=152) of the patients had a serum agglutination test (SAT) titer of ≥1:160. A blood culture was positive in 52 (34%) of the 111 patients tested. The most frequently prescribed regimen was rifampicin + co-trimoxazole in 81 (52.9%) patients. Relapse occurred in a small proportion (4.6%, n=7), and the majority (95.4%, n=146) had a complete remission.

Conclusions

The main route of transmission was the ingestion of unpasteurized dairy products. Brucellosis had a wide range of clinical presentation, involving multiple organ systems. Neurobrucellosis was the most frequent complication. The SAT was the most useful and reliable test for the diagnosis of brucellosis. Most patients were successfully treated with rifampicin and co-trimoxazole for six weeks.

## Introduction

Brucellosis is globally considered the most widespread zoonotic infection [1]. It is a significant threat to the public health system in many countries in North Africa and the Middle East, including Saudi Arabia [1,2]. *Brucella* species causing brucellosis are facultative intracellular, gram-negative coccobacilli [3]. Out of all the identified *Brucella *species, *B. abortus*, *B. melitensis,* and *B. suis* are the most contributing species to human illness, with *B. melitensis* being the most virulent [4,5]. Subclinical infection due to *Brucella *is mostly attributed to *B. abortus* [4]. The methods used to differentiate between the *Brucella *species are the production of urease and H_2_S, cell wall antigens, phage sensitivity, and dye sensitivity [4]. Reservoirs of the disease are food-producing animals such as goats, sheep, cattle, camels, and horses [6]. The prevalence of brucellosis in livestock varies globally, but the overall prevalence of brucellosis in livestock in Saudi Arabia is estimated at 17.4%, with an estimated animal infection rate of 26.1% in the Al-Qassim and Riyadh regions [7]. Brucellosis is transmitted from animals to humans through the ingestion of infected animal products such as unpasteurized milk and milk products and via contact with the tissue of infected animals, including blood, urine, aborted animal fetuses, and the placenta. Aerosols of infected animals can also cause human disease, and it is suggested that outbreaks are a result of aerosol inhalation occurring in animal stables [8]. The most common *Brucella *species causing human disease globally is *B. melitensis* [3]. An epidemiological study conducted in 2019 indicated that most of the human illness in Saudi Arabia is caused by *B. melitensis*, followed by *B. abortus* [9]. Human infection is prevalent in many parts of the world, including the Middle East and North Africa, Central Asia, Latin America, and the Mediterranean countries [10]. Brucellosis is considered an endemic disease in Saudi Arabia, with an infection rate of 70 per 100,000 people [11]. 

Patients with brucellosis usually present with non-specific symptoms such as malaise, weight loss, fever, chills, joint pain, and lymphadenopathy, all of which indicate a systemic involvement of the disease. In children, the main symptom at presentation is excessive sweating, followed by bone aches and chills. The main signs observed in children with proven brucellosis are arthritis and hepatomegaly [12]. Brucellosis is diagnosed with a blood culture, which takes a week or more. A bone marrow culture has a higher yield compared to blood culture. The most frequently used method of diagnosis in endemic areas is a standard agglutination test. Laboratory testing of patients with brucellosis may show anemia, leukopenia, and pancytopenia, secondary to bone involvement, as well as elevated inflammatory markers such as erythrocyte sedimentation rate (ESR) and C-reactive protein (CRP). X-ray imaging of the spine may indicate disc space narrowing, sclerosis, and bone damage in patients with profound bone involvement. In addition, a liver biopsy may demonstrate inflammation and the formation of granulomas [5]. The morbidity rate of brucellosis in the Saudi population is still increasing in different parts of the country, especially in rural regions. It is reported that the estimated prevalence of human infection in Saudi Arabia varies from 1.6% to 2.6%, affecting both genders in all age groups [13]. In the current study, we aimed to assess the epidemiological and clinical features, laboratory findings, treatment modalities, complications, and outcomes in children with brucellosis.

## Materials and methods

This retrospective chart review was conducted at King Abdulaziz Medical City (KAMC), in Riyadh, Saudi Arabia, a tertiary academic hospital acting as the primary referral institute for pediatric patients of the whole country. The study included all pediatric patients, 14 years or younger, diagnosed with brucellosis at King Abdullah Specialist Children’s Hospital from January 2015 to January 2019. The BESTCare electronic system was used to access patients’ medical records to extract the demographic data in terms of age, gender, family history, area of residence, history of consumption of raw milk or milk products, exposure to animal breeding, symptoms at presentation, and physical examination ﬁndings. The results of laboratory tests at presentation included complete blood count (CBC), erythrocyte sedimentation rate (ESR), C-reactive protein (CRP), aspartate aminotransferase (AST), alanine aminotransferase (ALT), serum agglutination test (SAT) titer, and the results of the blood culture were all obtained. The radiological features and the drug regimen used in the initial treatment and duration of therapy were also assessed. Patients diagnosed with neurobrucellosis, osteomyelitis, sacroiliitis, and spleen abscess were categorized as complicated brucellosis. Brucellosis relapse, which is “the recurrence of signs and symptoms of illness, with or without the presence of bacteria in blood after the period of treatment” [14] was also documented. The diagnosis of brucellosis was based on compatible signs and symptoms with a positive serology titer of ≥1:160 or a blood culture positive for *Brucella *spp. A positive blood culture in an asymptomatic patient is also diagnostic. All the data were entered in Microsoft Excel 2010 (Microsoft, Redmond, WA, USA) and analyzed by Statistical Package for Social Sciences (SPSS) Statistics version 23 (IBM Corp., Armonk, NY, USA).

The study was approved by the Institutional Review Board of King Abdullah International Medical Research Center, Ministry of National Guard-Health Affairs, Riyadh, Kingdom of Saudi Arabia (approval number RC20/267/R). Patient confidentiality was ensured, and the patients' data were collected and used by the research team only. Serial numbers were used instead of medical record numbers to ensure anonymity. Due to the retrospective nature of the study, and the use of anonymized patient data, the requirement for informed consent was waived.

## Results

The sample size was 153 patients and all were included in the analysis. The mean age was 7.75 ± 3.28 years with male predominance (69.6%, n=107). The demographic information is displayed in Table 1. The majority of the patients (93.5%) were from the Riyadh region. Ingestion of unpasteurized dairy products, especially camel milk, was the most frequent risk factor for transmission observed in 51 (33.3%) patients. Direct animal contact was documented in seven (4.6%) patients. No risk factor for transmission was identified in 95 (62%) patients. A family history of brucellosis was observed in 39 (25.5%) patients.

**Table 1:** Demographic information SD: standard deviation

Age category	Male (n=107)	Female (n=46)	Entire cohort (n=153)
0-2 years	7	2	9 (5.9%)
3-6 years	30	19	49 (32%)
7-10 years	66	16	82 (53.6%)
11-14 years	4	9	13 (8.5%)
Age mean ± SD	8.06 ± 3.28	7.16 ± 3.13	7.75 ± 3.28

Presentation

The majority (79.7%, n=122) presented with constitutional symptoms. The most frequent presenting symptom was fever (77.12%, n=118). Less than half (41.2%, n=66) of the patients presented with musculoskeletal symptoms, particularly arthralgia. The remaining symptoms are summarized in Table 2. A small proportion (3.9%, n=6) had complicated brucellosis with neurobrucellosis as the most frequent complication. Hospitalization for brucellosis was required in 23 (15%) patients, with 130 (85%) followed-up as outpatients.

**Table 2:** Frequency of symptoms by organ system

Symptoms	n (%)
Constitutional Symptoms	122 (79.7)
Fever, fatigue, headache, night sweats, tiredness, weight loss, failure to thrive	
Gastrointestinal symptoms	11 (7.2)
Abdominal pain, diarrhea, vomiting, dysphagia	
Musculoskeletal symptoms	63 (41.2)
Joint pain (knee, hip, shoulder, ankle), muscle pain, limited joint range of motion	
Neurological symptoms	6 (3.9)
Limb weakness, slurred speech, visual disturbances	
Respiratory symptoms	10 (6.5)
Cough, nasal congestion, throat congestion, sore throat	
Dermatological symptoms (generalized rash)	1 (0.7)
Asymptomatic	10 (6.5)

Laboratory findings

Leukocytosis was observed in 17 (11.11%) patients, leukopenia in seven (4.57%), and thrombocytopenia in 13 (8.49%). The ESR was elevated in 101 (66%) patients, and the CRP in 43 (28.1%). The AST and ALT levels were high in 67 (43.79%) and 15 (9.8%) patients, respectively. The total number, minimum, maximum, and mean of the laboratory findings are provided in Table 3. The SAT titers at presentation and the end of treatment are illustrated in Table 4.

**Table 3:** Laboratory findings RBC: red blood cell; WBC: white blood cell; ESR: erythrocyte sedimentation rate; CRP: C-reactive protein; AST: aspartate aminotransferase; alanine aminotransferase

	n	Minimum	Maximum	Mean ± standard deviation
RBC	145	3	45	5.02 ± 3.37
WBC	147	1.8	20.4	7.65 ± 2.84
Platelets	146	22	659	284.32 ± 102.24
ESR	123	2	517	40.04 ± 49.9
CRP	55	1	126	25.11 ± 27.1
AST	110	18	167	45.64 ± 27.1
ALT	110	5	184	32.82 ± 27.3

**Table 4:** SAT titers of brucellosis patients at presentation and end of treatment SAT: serum agglutination test

	Brucella melitensis		Brucella abortus	
SAT titers	Presentation	End of treatment	Presentation	End of treatment
	n (%)	n (%)	n (%)	n (%)
<1:160	1 (0.65)	11 (7.19)	1 (0.65)	14 (9.15)
1:160	2 (1.31)	41 (26.8)	11 (7.19)	50 (32.68)
1:320	11 (7.19)	28 (18.3)	19 (12.42)	21 (13.73)
1:640	28 (18.3)	23 (15.03)	18 (11.76)	21 (13.73)
1:1280	27 (17.65)	20 (13.07)	29 (18.95)	20 (13.07)
1:2560	24 (15.69)	9 (5.88)	23 (15.03)	6 (3.92)
1:5120	22 (14.38)	4 (2.61)	17 (11.11)	1 (0.65)
1:10240	13 (8.5)	1 (0.65)	10 (6.54)	1 (0.65)
1:20480	10 (6.54)	1 (0.65)	11 (7.19)	0
>1:20480	14 (9.15)	1 (0.65)	14 (9.15)	5 (3.27)
Total	152 (99.35)	139 (90.85)	153 (100)	139 (90.85)

Diagnosis

The vast majority (99.35%, n=152) of the sample had a SAT titer of ≥1:160, and one (0.65%) patient <1:160. This seronegative patient had a positive cerebrospinal fluid (CSF) SAT titer for *Brucella *and was later diagnosed with neurobrucellosis. Blood cultures were taken from 111 (72.6%) patients and were positive in 52 (34%), and negative in 59 (38.6%) patients. Less than a third (27.5%, n=42) of the sample did not have a blood culture. 

Treatment regimen

The duration of treatment in the majority (64.7%, n=99) of the sample was six weeks. A small proportion (13.8%, n=21) required more than six weeks of treatment. The remaining patients required less than six weeks of treatment. The most frequently used drug regimen was rifampicin with co-trimoxazole in 81 (52.9%), followed by rifampicin with doxycycline in 54 (35.3%) patients. Other drug regimens used are listed in Table 5. A small proportion (4.6%, n=7) relapsed, and the majority (95.4%, n=146) had a complete remission.

**Table 5:** Antibiotic regimen administered for brucellosis patients

Regimen	Frequency	Percentage (%)
Doxycycline	1	0.65
Doxycycline + co-trimoxazole	5	3.27
Rifampicin + ciprofloxacin	3	1.96
Rifampicin + co-trimoxazole	81	52.94
Rifampicin + doxycycline	54	35.29
Rifampicin + gentamicin + co-trimoxazole	3	1.96
Rifampicin + gentamicin + doxycycline	3	1.96
Rifampicin + co-trimoxazole + doxycycline	2	1.31
Not recorded	1	0.65
Total	153	100

## Discussion

Brucellosis represents a major health concern in Saudi Arabia and other Middle Eastern countries. It remains one of the most frequent zoonotic infections in the Arabian Peninsula and globally [15]. Brucellosis may cause serious illness in all age groups. The main modes of transmission include the consumption of contaminated raw milk or milk products, contact with products of infected animals such as blood or urine, and contact with the placenta of infected animals [8]. In the current study, the main route of transmission was the ingestion of unpasteurized dairy products. It is believed that infection with *B. abortus* is rare in children, as reported by Mantur et al. [16]. However, *B. abortus* is not uncommon in Saudi children as both *B. melitensis* and *B. abortus* were detected in all the children in this study. The majority of brucellosis cases in the current study occurred in males, similar to Iranian and Turkish children [2,17]. The fact that boys are more involved in animal care may be a reasonable explanation for the male predominance in brucellosis infection [18]. More than half of the sample were in the six to 12 years age group. The lowest infection rate was recorded in children less than two years and older than 12 years with a mean age of 7.75 years. In the current study, children younger than two years were the least infected age group. A possible reason is a lower exposure to raw milk and milk products compared to children in older age categories [16]. Nearly 25% of the sample had a positive family history in which at least one family member also had brucellosis infection, compared to 41% in a study of Greek children [19]. This difference may be secondary to insufficient documentation as nearly 25% of the sample did not have a complete medical record in terms of family history.

The majority of the cases presented had uncomplicated brucellosis and presented with constitutional symptoms such as fever, fatigue, and musculoskeletal symptoms, such as joint pain. The presenting symptoms varied as many organ systems, including the digestive, respiratory, and nervous systems were involved. Vomiting and diarrhea were the most prevalent gastrointestinal symptoms, followed by abdominal pain, in only two patients. Respiratory symptoms included throat congestion and cough. A dermatological presentation of brucellosis is generally rare [20,21]. However, a four-year-old boy with a history of ingestion of unpasteurized dairy products presented with a generalized rash that was later attributed to brucellosis. Although the vast majority of the sample presented with symptoms, an asymptomatic presentation occurred in 10 patients who required pharmacological treatment after confirming the diagnosis of brucellosis with an SAT titer of ≥1:160. Some of the asymptomatic patients were diagnosed due to screening recommendations as a family member had brucellosis. Two-thirds of the patients had an unremarkable or normal physical examination on presentation. However, the physical examination of some patients indicated a limited range of motion in a single joint, with the knee joint being the most affected. Other less frequently affected joints included the hip, ankle, and shoulder.

According to literature, laboratory findings in an active *Brucella *infection are not useful in creating differential diagnoses, as many are nonspecific [22,23]. In the current study, the ESR was considered an important laboratory finding that was used to assess the patient’s response to the medication and to evaluate the severity of the infection. The CRP was also nonspecific, but an important marker used for the same purpose. About half of the sample presented with elevated liver enzymes, particularly AST. Elevated liver enzymes are a frequent finding in brucellosis; however, the clinical significance is questionable.

For the diagnosis of brucellosis, a potential exposure, clinical features suggesting a *Brucella *infection, and serological tests with or without a positive blood culture are required. Isolating the *Brucella *spp. from the blood, bone marrow, or other tissue fluids is the gold standard for diagnosis. However, a blood culture has a low sensitivity and is not required for the diagnosis of brucellosis. Of the sample (n=153), only 52 (34%) had a positive blood culture, and 59 (38.6%) a negative blood culture. The remaining 42 (27.5%) patients did not require a blood culture to confirm the diagnosis. Serological tests are the main test in the diagnosis of brucellosis. An SAT titer of ≥1:160 is suggestive of active *Brucella *infection. Although having a low specificity, serological tests provide a sensitivity of 65% to 95% for the diagnosis of brucellosis.

Some complications due to brucellosis were observed in the present study, including neurobrucellosis, sacroiliitis, osteomyelitis, and a splenic abscess. Neurobrucellosis was identified in three (1.9%) patients. All three patients presented at the Emergency Department with neurological symptoms. The first patient had a relapsed *Brucella *infection after a complete recovery confirmed with a negative serological SAT titer. He presented to the ER complaining of right upper limb weakness, confusion, headache, and abnormal speech, later diagnosed as Broca’s aphasia. His serological and CSF SAT titers were positive at ≥1:160 and 1:20 respectively. Both CSF and blood cultures were negative for *Brucella*. The CSF analysis indicated lymphocytosis, normal protein, and high glucose. A computed tomography (CT) scan showed left basal ganglia and left frontal lobe focal hypodensities, secondary to a middle cerebral artery (MCA) ischemic stroke, proven by magnetic resonance imaging (MRI). The second patient presented with fever, headache, and neck pain suggesting meningeal irritation. Both his serological and CSF SAT titers were positive for *Brucella *with the CSF titer critically high (1:160). However, blood and CSF cultures were negative. The CSF analysis revealed lymphocytosis, high protein, and low glucose levels. A CT scan showed mild ventricular dilatation and focal right centrum semiovale hypodensity, and the MRI indicated anterior dural and leptomeningeal enhancement. The third patient experienced two episodes of generalized tonic-clonic seizures and presented with hallucination, dysarthria, and ataxia. Despite her symptoms, serum and CSF cultures were negative for *Brucella *along with serum SAT titers but positive CSF SAT titers (1:10). The CSF analysis demonstrated lymphocytosis, high protein, and glucose levels. An MRI of the brain showed diffuse leptomeningeal enhancement suggestive of meningitis. The diagnosis of neurobrucellosis was made as a result of positive neurological symptoms and a positive CSF SAT titer for *Brucella*, after excluding all other potential causes. All patients had negative CSF and blood cultures, regardless of having neurobrucellosis. Imaging studies confirmed the diagnosis and showed different patterns of central nervous system (CNS) involvement in the three patients. All patients were treated as inpatients with IV antibiotics and discharged with oral antibiotics. Details are shown in Table 6.

**Table 6:** Laboratory findings and treatment plan of the neurobrucellosis patients CSF: cerebrospinal fluid; RBC: red blood cell; WBC: white blood cell; SAT: serum agglutination test; IV: intravenous

Findings	Patient 1	Patient 2	Patient 3
CSF protein (mg/dl)	0.33	3.03	0.70
CSF glucose (mg/dl)	8.60	1.60	6.80
CSF appearance	clear	clear	turbid
CSF color	colorless	colorless	xanthochromic
CSF RBC (per mm^3^)	<1	3	5265
CSF WBC (per mm^3^)	11	11	10
CSF lymphocytes (%)	96	80	87
CSF monocytes (%)	4	7	5
Brucella CSF SAT titer	1:20	1:160	1:10
Brucella CSF culture	negative	negative	negative
B. melitensis blood SAT titer	1:640	1:1280	<1:160
B. abortus blood SAT titer	1:1280	1:1280	<1:160
Brucella blood culture	negative	negative	negative
Inpatient treatment plan	IV rifampicin + IV doxycycline + IV ceftriaxone	rifampicin + doxycycline + co-trimoxazole (all orally)	IV vancomycin + IV ceftriaxone + IV levetiracetam + IV rifampicin + IV doxycycline
Outpatient treatment plan and duration	Oral rifampicin + doxycycline for 9 weeks	Continued the same regimen orally for 9 weeks	Oral rifampicin + co-trimoxazole for 9 weeks

A 10-year-old boy with a history of raw milk ingestion presented with a 10-day history of hip pain which initially started on the left side and then became bilateral. This pain caused a significant limitation in the range of motion and increased in both active and passive movements. *Brucella *serological tests were requested but were taking a prolonged time, and with a high index of suspicion, the patient was prescribed oral doxycycline and co‐trimoxazole empirically. An MRI of the hip joint revealed a small abscess, which was suggestive of sacroiliitis secondary to brucellosis. The *Brucella *SAT titers were positive and highly elevated. The patient discontinued the oral co‐trimoxazole and rifampicin, doxycycline, and gentamicin were prescribed intravenously. He was later discharged with oral rifampicin and doxycycline for five weeks. His *Brucella *titers and inflammatory markers reduced significantly in the next follow-up with a complete resolution of the infection.

A seven-year-old boy with a positive history of raw milk ingestion presented with a two-week history of fever and unilateral ankle pain and swelling. He was initially managed with oral antipyretics, analgesics, and diclofenac topical gel, which resulted in a significant reduction in fever and ankle pain. However, he presented again to the ED with fever and ankle swelling. An X-ray of the ankle was done which indicated a mild joint effusion. Distal tibial osteomyelitis, secondary to *Brucella *infection was suspected due to his positive history of raw milk ingestion, which was later confirmed with a positive blood culture and serology. The patient was successfully treated with oral doxycycline and rifampicin for six weeks.

A nine-year-old boy presented with a one-month history of fever. He had a congested throat twice during the month and was treated with antibiotics and antipyretics in a private clinic. His symptoms improved initially, but his fever returned two days later. His family indicated that he consumed raw milk recently, which raised suspicion for brucellosis, particularly as some of his family members were previously diagnosed with the disease. Hepatosplenomegaly was noted during the physical examination and his blood tests showed pancytopenia and elevated liver enzymes. An ultrasound and CT scan of the abdomen showed multiple splenic abscesses. A bone marrow aspiration was performed to rule out malignancy, which indicated the presence of lymphohistiocytic granulomatous changes consistent with *Brucella *infection. Serological tests confirmed the diagnosis of brucellosis and the patient was successfully treated with oral doxycycline and rifampicin for six weeks with a rapid improvement in cell count.

The observed relapse rate in the present study was lower than reported in the literature (4.6%). In Turkey, a study retrospectively analyzing 90 pediatric brucellosis cases reported a relapse rate of 6.6%. The relapsed cases were effectively treated with triple‐drug regimens and had a low sequelae rate [2]. In Iran, a study assessing the clinical, laboratory, and epidemiologic characteristics of pediatric brucellosis, reported a relapse rate of 21%. It is worth noting that the most frequently used drug regimen was combined trimethoprim-sulfamethoxazole (co-trimoxazole) and rifampicin [17]. In Israel, a study comparing the outcome of four antimicrobial regimens reported an overall relapse rate of 20%. All the relapsed patients recovered after the second course of antibiotic therapy [24]. In the Republic of Macedonia, a study assessing the outcomes of osteoarticular brucellosis reported a relapse rate of 13.8% [25], and a second Macedonian study, with 317 pediatric brucellosis cases, reported a relapse rate of 6.6% [26]. In the United States, a nonendemic region, a study conducted in Dallas, Texas, found a relapse rate of 25% [27]. In Saudi Arabia, a study conducted in the southwestern city of Najran reported a relapse rate of 3.5%, lower than the present study [28]. In Riyadh, a study retrospectively analyzed 115 pediatric brucellosis cases that occurred from 1984 to 1995, reported a relapse rate of 9%, higher than the present study [29].

There have been conflicting reports in the literature related to the treatment modality of choice in childhood brucellosis. In the present study, the most frequent treatment regimen was rifampicin and co-trimoxazole (52.9%), frequently for six weeks (64.7%). These findings support a Turkish study, reporting that the treatment of childhood brucellosis with co‐trimoxazole and rifampicin was effective with a low relapse rate [2], supporting the current study with a relapse rate of 4.6%. In the Iranian study, the most frequent treatment regimen was also co-trimoxazole and rifampicin, however with a relapse rate was 21% [17]. In the Najran study, rifampicin, prescribed as a monotherapy, was the treatment of choice. The relapse rate was 3.5% [28]. The Israeli study, however, stated that monotherapy for childhood brucellosis was associated with higher relapse rates. In addition, they reported that a longer treatment duration, reduced the chances of relapse [24].

There were some limitations to the current study. Due to its retrospective nature, cases were not directly observed by the research team, and missing data could not be retrieved. Some patient data was not completely documented in the BESTCare system, such as the history of consumption of raw milk or milk products. This may result in inaccurate results. Lastly, recall bias in the children’s caregiver may have resulted in bias in terms of the signs and symptoms and an incomplete record.

## Conclusions

In conclusion, most brucellosis cases in the current study were males aged seven to 10 years. The main route of transmission was the ingestion of unpasteurized camel dairy products. Infection with *B. abortus* is not uncommon in Saudi children. Brucellosis has a wide range of clinical presentation, involving multiple organ systems. Neurobrucellosis was the most frequent complication, however complicated cases were generally rare. Erythrocyte sedimentation rate was the most important laboratory finding used to assess the patient’s response to medication. Serum agglutination test was the most useful and reliable test for the diagnosis of brucellosis. Almost all patients responded well to the combination therapy with at least two antibiotics. The most frequent treatment regimen was rifampicin and co-trimoxazole for six weeks.
